# Prevention of the Recurrence of Anaemia in Gambian Children Following Discharge from Hospital

**DOI:** 10.1371/journal.pone.0011227

**Published:** 2010-06-21

**Authors:** Kalifa A. Bojang, Paul J. M. Milligan, David J. Conway, Fatou Sisay-Joof, Muminatou Jallow, Davis C. Nwakanma, Ismaela Abubakr, Fanta Njie, Brian Greenwood

**Affiliations:** 1 Medical Research Council Laboratories, Banjul, The Gambia; 2 London School of Hygiene and Tropical Medicine, London, United Kingdom; Mahidol University, Thailand

## Abstract

**Background:**

In malaria endemic countries, children who have experienced an episode of severe anaemia are at increased risk of a recurrence of anaemia. There is a need to find ways of protecting these at risk children from malaria and chemoprevention offers a potential way of achieving this objective.

**Methods:**

During the 2003 and 2004 malaria transmission seasons, 1200 Gambian children with moderate or severe anaemia (Hb concentration <7 g/dL) were randomised to receive either monthly sulfadoxine-pyrimethamine (SP) or placebo until the end of the malaria transmission season in which they were enrolled, in a double-blind trial. All study subjects were treated with oral iron for 28 days and morbidity was monitored through surveillance at health centres. The primary endpoint was the proportion of children with moderate or severe anaemia at the end of the transmission season. Secondary endpoints included the incidence of clinical episodes of malaria during the surveillance period, outpatient attendances, the prevalence of parasitaemia and splenomegaly, nutritional status at the end of the malaria transmission season and compliance with the treatment regimen.

**Results:**

The proportions of children with a Hb concentration of <7 g/dL at the end of the malaria transmission season were similar in the two study groups, 14/464 (3.0%) in children who received at least one dose of SP and 16/471 (3.4%) in those who received placebo, prevalence ratio 0.89 (0.44,1.8) P = 0.742. The protective efficacy of SP against episodes of clinical malaria was 53% (95% CI 37%, 65%). Treatment with SP was safe and well tolerated; no serious adverse events related to SP administration were observed. Mortality following discharge from hospital was low among children who received SP or placebo (6 in the SP group and 9 in the placebo group respectively).

**Conclusions:**

Intermittent treatment with SP did not reduce the proportion of previously anaemic children with moderate or severe anaemia at the end of the malaria season, although it prevented malaria. The combination of appropriate antimalarial treatment plus one month of iron supplementation and good access to healthcare during follow-up proved effective in restoring haemoglobin to an acceptable level in the Gambian setting.

**Trial Registration:**

ClinicalTrials.gov NCT00131716

## Introduction

Anaemia remains one of the most important health problems for children in malaria-endemic countries of Africa; the prevalence of severe anaemia, usually defined as a Hb<5g/dL, among children in the community varies from 1.3% to 6.4% [1]–[6] and moderate anaemia (Hb<7g/dL) is frequent. Severe anaemia is a major cause of hospital admission and contributes substantially to inpatient paediatric mortality and mortality. Among children admitted to hospital in sub-Saharan Africa the prevalence of severe anaemia ranged from 7% to 29% in different epidemiological settings [1]–[6]. The case fatality rate (CFR) for children with severe anaemia is estimated to be between 8% and 18% [3], [4], [6]–[10]. Causes of severe anaemia in African children include nutritional deficiencies, infections, and haemoglobinopathies. However, infection with *Plasmodium falciparum* is one of the main causes in malaria endemic areas. Malaria infection results in destruction of both infected and non-infected red blood cells and depresses the ability of the bone marrow to produce new red blood cells.

Studies in a high malaria transmission area of western Kenya indicated that children treated for severe anaemia (Hb<5g/dL) were at high risk of dying after they had been discharged from hospital due to a rebound of their anaemia [11], [12]; 14%–16% of children treated for severe malaria died at home within eight weeks of discharge. Ineffective antimalarial treatment and persistent parasitaemia on discharge from hospital were strong predictors of subsequent out-of-hospital mortality. In these settings, effective antimalarial treatment and the targeted delivery of appropriate malaria control interventions may be a cost-effective approach to reducing the morbidity and mortality associated with severe anaemia. These findings indicate that there is a need to improve the management of African children with severe anaemia both within hospital and after their discharge. Prevention of malaria through provision of an insecticide treated bednet (ITN) is likely to be one effective option. Chemoprevention offers another potential approach.

Intermittent preventive treatment (IPT), which involves administration of a full treatment course of an antimalarial at specified times to at risk subjects regardless of whether or not they are known to be parasitaemic, was devised as a strategy for overcoming some of the problems associated with chemoprophylaxis whilst taking advantage of its protective effects. IPT was developed initially for use in pregnant women and then adapted to malaria control in infants (IPTi) and older children (IPTc). The role of IPT in the prevention of malaria and anaemia in children has been evaluated in a number of trials. In Mali, an area of seasonal malaria transmission, two doses of sulphadoxine-pyrimethamine (SP) given to children aged 6 months to 9 years at an interval of two months gave a protective efficacy of 40% against clinical attacks of malaria [13]. In another study undertaken in Niakhar, Senegal, an area of intense but short seasonal malaria transmission, SP and one dose of artesunate (AS) given to children less than 5 years old three times at one monthly intervals throughout the peak period of malaria transmission reduced clinical attacks of malaria by 86% [14]. A further trial was undertaken in the same study site to compare different treatment regimens and high levels of efficacy against episodes of clinical malaria were observed with each treatment regimen [15]. In another comparative study carried out in Ghana, monthly amodiaquine (AQ) plus AS was compared with bimonthly AQ plus AS and bimonthly SP. Monthly AS plus AQ was the most effective regimen, giving 69% protection against clinical episodes of malaria [16]. These results suggest that IPTc has potential as an affordable malaria control tool. However, the potential role of IPT in the prevention of a recurrence of severe anaemia in children who have already been treated for moderate or severe anaemia ( Hb<7g/dl) remains to be established. Thus, we have investigated whether monthly IPT with SP given during the malaria transmission season can protect Gambian children treated previously for moderate or severe malaria in hospital against a recurrence of anaemia.

## Methods

**Figure 1:**
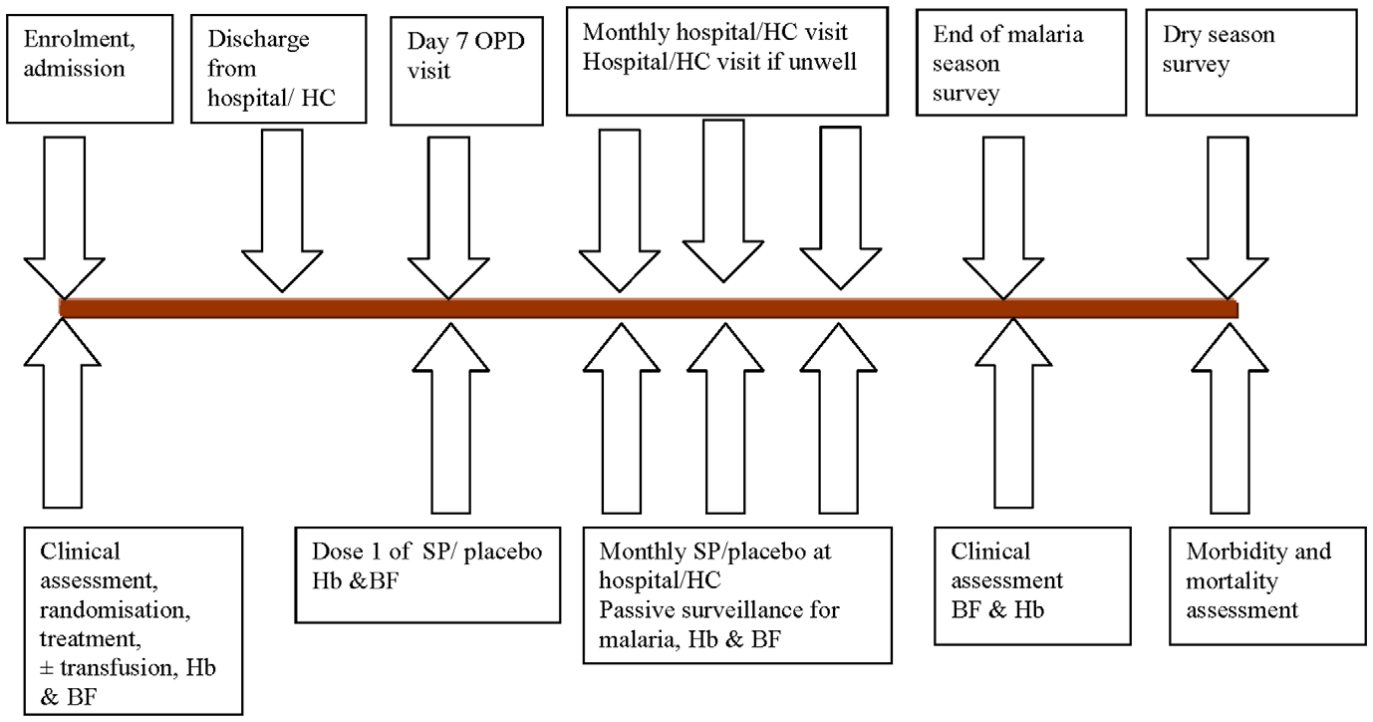
Overall study design. The duration of follow up varied depending upon the time in the malaria transmission season when a child was recruited. OPD = outpatient department; Hb = haemoglobin; BF = blood film.

The protocol for this trial and supporting CONSORT checklist are available as supporting information; see Checklist S1 and Protocol S1. A summary of the recruitment and follow-up procedures is shown in Fig. 1.

### Objectives

The primary objective of the study was determination of the impact of IPT with SP on the proportion of children previously treated for severe or moderate anaemia who had a recurrence of their anaemia at the end of the malaria transmission season. Secondary objectives included study of the impact of IPT with SP on the incidence of clinical episodes of malaria during the surveillance period, outpatient attendances, the prevalence of parasitaemia and splenomegaly and nutritional status at the end of the malaria transmission season and compliance with the treatment regimen.

### Study area and population

The study was conducted in the Western Region of The Gambia, which includes the capital Banjul. Transmission of malaria in The Gambia is seasonal. The climate is typical of sub-Sahelian Africa with a long dry season, which lasts from December–June, followed by a relatively short rainy season from July–November during which most malaria transmission occurs. Morbidity and mortality from malaria follow this pattern, both occurring much more frequently during the rainy season, with a peak in the month of October. The annual entomological inoculation rate varies enormously across the country and is in the range of 1–177 infected bites per person year [17]. During the course of the study, first line treatment for uncomplicated malaria in The Gambia changed from chloroquine to the combination of chloroquine and SP. Since 2008, the recommended first line treatment for uncomplicated malaria has changed to artemether-lumefantrine (Coartem™, Norvatis Pharma, Basel Switzerland). In 2001, the PCR-corrected treatment failure rate in symptomatic children at day 28 after the start of treatment with SP was 6% [18]. At the time of the study there were no standardized guidelines for the prevention of anaemia in children.

1200 children were recruited from the Royal Victoria Teaching Hospital (RVTH), Banjul, the Medical Research Council (MRC) Hospital, Fajara, or from the major health centres at Brikama, Essau and Faji Kunda, 600 children during the 2003 transmission season (July to December), and another 600 children in the 2004 malaria transmission season. The study was extended to include the WEC clinic, Sibanor, in 2004.

### Screening, enrolment and randomization

The trial was designed as an individually randomised, controlled, double-blind study. Children were recruited on presentation to the out-patient clinic (OPD) or ward of one of the participating health facilities. Children suspected of having anaemia and who were in the right age group had their Hb concentration measured and a blood film examined for the presence of malaria parasites. Children were eligible for enrolment if they were aged between 3 months and 9 years and had a Hb concentration <7 g/dL. Children who did not meet these criteria were excluded. Individual, signed consent was obtained from the parents or guardians of children who met the study inclusion criteria. The randomisation list was generated by a senior MRC statistician. Provided that consent was given, children were individually randomized into either the SP or the placebo group in a 1∶1 ratio at the time of admission, using permuted blocks of 12 generated by computer using the STATA program. Blocks were not split across centres. Tablets (enough for 6 doses) were packed into envelopes bearing the randomization number by MRC staff not involved in the trial in any other way. The next envelope in sequence was assigned to the child at the time of their admission to hospital. Copies of the randomization code, kept in a sealed opaque envelope, were held by the local safety monitor and the MRC Laboratories accountant. Some additional replacement envelopes were also prepared, which were separately numbered, with a listing indicating which of these envelopes should be used in the event that a child's envelope was lost or damaged, None of the investigators, health care centre staff or laboratory staff participating in the trial had access to the code during the trial. A formal attempt to demonstrate blinding (by asking the subjects to guess their treatment allocation) was not undertaken. The code was provided to the investigators only after a locked copy of the database had been given to the chairman of the Data Safety and Monitoring Board.

### Initial case management

On admission, children were clinically evaluated and additional investigations, for example lumbar puncture, were undertaken when these were indicated on clinical grounds. The majority of children with malaria were treated with intramuscular quinine followed by SP. However, a small number received chloroquine plus SP. Patients were treated for other conditions as clinically indicated. Children were kept in hospital until all signs of respiratory distress had subsided and until their Hb concentration had increased over that found on admission. Their average duration of stay in hospital was 4 days (range 1 to 36 days). Children with a haemoglobin concentration of <5 g/dL and/or signs of respiratory distress were scheduled for immediate transfusion with whole blood in accordance with national guidelines. All patients received iron for 28 days (ferrous fumerate syrup at a target dose of 2mg/kg), starting at the time of their discharge from hospital. Each child was provided with a photo ID card with their unique study number prior to their discharge from hospital. Children were asked to return for follow-up 7 days after discharge. At this visit, a fingerpick blood sample was collected for Hb concentration measurement and thick blood film examination for malaria parasites. Any medical condition detected during the visit was treated appropriately.

### Chemoprevention

The first dose of trial medication was given under supervision by project staff at the first follow-up visit to the hospital or health centre where the child had been admitted, scheduled to be seven days after their discharge from hospital. The envelope containing the trial medication bearing the child's study number was then transferred to the health centre closest to the child's home where the patient continued to receive monthly chemoprevention given by trained field workers. Subsequent monthly doses of SP or placebo were given under the supervision of clinic staff until the end of the malaria transmission season. Thus, the number of treatments that a child was expected to receive varied depending upon the time of the year at which they were recruited. SP tablets (500 mg sulphadoxine/25 mg pyrimethamine) (Cosmos Ltd., Nairobi, Kenya) were given at an approximate dose of 1.25 mg pyrimethamine/25 mg sulphadoxine per kg. Placebo tablets, which were identical in shape and colour to the SP tablets, contained mainly lactose and maize starch. Older children took the study drugs as tablets, which were swallowed whole or chewed. Younger children were given their tablets crushed, suspended in water and administered with a spoon.

### Morbidity surveillance during the rainy season

Mothers/guardians were encouraged to take their child to one of the participating health facilities at any time after discharge from hospital if their child became unwell. In order to facilitate OPD visits, transport fares were given to mothers each time a study subject reported to a health facility. Using this method of passive surveillance, which best reflects the likely public health impact of an antimalarial intervention, it was unlikely that a significant number of cases of severe malaria or other conditions would have been missed during the study but a larger number of cases of milder malaria might have been identified using active surveillance with home visits. A finger prick blood sample was obtained from any study child who presented with an axillary temperature ≥37.5°C or a history of fever within the previous 48 hours for preparation of a thick blood film for microscopy and blood spots on a glass fibre mat (Wallac, Finland) were obtained for parasite genotyping. Diagnosis and treatment were recorded in a standardized form each time a study child was seen at a health facility. Children with documented fever (axillary temperature of ≥37.5°C) or a history of recent fever and malaria parasitaemia were treated with SP and chloroquine in accordance with Gambia Government treatment guideline. Children who presented with severe malaria were treated with IM quinine and those who presented with uncomplicated malaria within one week of receiving SP chemoprevention received oral quinine. During follow-up, children with a haemoglobin concentration <9 g/dL were treated with iron for a further 28 days if they have completed their initial iron treatment and those with severe anaemia were referred for admission. Deaths that occurred at home were investigated using the post-mortem questionnaire technique and the cause of death established whenever possible.

### Adverse events

All adverse reactions which might be related to drug administration were documented at each contact with the study subject. Particular attention was paid to the presence of any skin reactions, in particular Stevens-Johnson syndrome as this is the most important adverse reaction related to SP administration.

### Cross-sectional surveys

All children were visited in the January after the end of the transmission season in which they were enrolled. A standardized questionnaire was administered to collect information regarding any illness that had occurred since the last visit, use of healthcare facilities and use of medicines. Information on the use of bednets was collected during this survey. Children were examined by a physician, anthropometric data were collected, and a finger-prick blood sample was obtained for preparation of a thick blood smear and determination of Hb concentration. At the end of the following dry season in May, study subjects were visited at home and a short questionnaire administered to document morbidity and mortality during the preceding five months.

### Laboratory methods

Thick smears were prepared in duplicate so that if the subject had symptoms of malaria, one smear could be stained with Field's stain and read promptly to guide treatment. The other smear was stained with Giemsa stain and 200 high power fields (HPF) were examined before a smear was declared negative. Only the Giemsa-stained slide readings were used for the trial analysis. Parasite density was expressed per µl with the assumption that 1 parasite per high-powered field (hpf) equals 500 parasites per µl [19]. All slides were read by two laboratory technicians. If there was disagreement between these readings on parasite positivity or if the difference of the log-densities recorded was more than 1.5, slides were read by a third technician. Agreement was reached after the slides had been re-checked. Discrepancies occurred mainly in smears with very low parasite densities. On rare occasions during follow-up, when a laboratory technician was not available to read a thick blood smear for malaria parasites, the Core™ Malaria Pf test (CORE Diagnostics, Birmingham, UK) was used to guide treatment and a thick blood smear collected for subsequent confirmation of the diagnosis.

Haemoglobin concentration was measured at recruitment, during morbidity surveillance and at the end of malaria transmission season surveys using a portable haemoglobinometer (HemoCue AB, Sweden).

DNA was extracted from glass fibre mats using the chelex technique [20]. Polymorphisms in the *pfdhfr* and the *pfdhps* genes, associated with resistance to pyrimethamine (codons 51, 59 and 108 of *dhfr*) and sulphadoxine (codons 437 and 540 of *dhps*) respectively, were tested for using PCR amplification followed by restriction fragment length polymorphism analysis (PCR-RFLP) [21].

### Statistical methods

The sample size was determined on the basis of previous studies carried out in The Gambia. We estimated that 20% of children would be moderately anaemic (PCV<20% - approximately equal to a Hb concentration of 7 g/dL) at the end of the malaria transmission season and that the attack rate of malaria (cumulative incidence of malaria) in the control group would be 0.1 episodes per month. To detect a reduction by one third (from 20% to 13%) in the prevalence of moderate to severe anaemia (Hb<7g/dL) at the end of the malaria transmission season with a power of 80%, using a significance level of 5% and assuming 15% loss to follow-up, 600 children were needed in each group. With this sample size, the study had 80% power using a 5% level of significance, to detect a reduction by one third in the incidence of clinical attacks of malaria in the treatment group. The primary endpoint was the prevalence of anaemia (Hb<7), the prevalence ratio was calculated with 95% confidence interval; the log-binomial model [22] was used to estimate the prevalence ratio adjusted for covariates (baseline Hb concentration, malaria infection at baseline, bednet usage and recruitment centre) which were specified in an analysis plan written before unblinding. In the primary analysis only children who received at least one dose of IPT or placebo, and were followed up, were included. Mean haemoglobin concentration was compared using a t-test, and an adjusted difference between means was obtained using normal regression. An episode of clinical malaria was defined as an illness accompanied by (a) an axillary temperature of ≥37·5°C or a history of fever within the previous 48 hours, (b) no other obvious cause for the fever and (c) the presence of *P falciparum* asexual stage parasitaemia at any density. Malaria with high density parasitaemia was defined as above but included only children with a *P falciparum* parasite density of ≥5000/µL. Malaria parasitaemia at the cross-sectional survey was defined as the presence of asexual stage *P. falciparum* parasitaemia of any density regardless of the presence or absence of the symptomatic criteria used for the definition of clinical malaria. A serious adverse experience was defined as any event which was fatal, life threatening, disabling or incapacitating or resulted in hospitalisation or prolonged a hospital stay or was associated with overdose (either accidental or intentional). Adverse events were not formally graded for severity. Cox regression was used to compare the incidence of malaria, and of anaemia, between the two groups. Efficacy was defined as the percentage reduction in the number of events in the intervention group, estimated as 100×(1-hazard ratio). All events were included in the analysis and a robust estimator of the variance was used to allow for the lack of independence among repeated episodes in the same child. For the malaria endpoint, estimates were adjusted for the effects of bednet usage defined as sleeping every night under an intact or impregnated net, recruitment centre and age. For anaemia, estimates were adjusted for net use, recruitment centre, baseline prevalence of parasitaemia, and baseline Hb concentration. Interaction between treatment group and bednet use was examined for both outcomes. Time at risk started at the beginning of the surveillance period (the date of the day 7 visit after discharge) and ended on the last day of December of the same year. If a subject was lost to follow-up observations were censored from the date of the first scheduled visit that they failed to attend. If a subject withdrew consent or died, the observation for that subject was censored at the date of the death or withdrawal. Children who moved temporarily out of the study area but returned to take some of the courses of treatment remained as part of the study cohort. Analyses were done using STATA version 10 (STATA Corporation, TX, US).

### Ethical review

The study was approved by the London School of Hygiene & Tropical Medicine Ethics Committee and by the joint MRC/Gambia Government Ethics Committee. The conduct of the trial was guided by a Data Safety and Monitoring Board.

## Results

**Figure 2:**
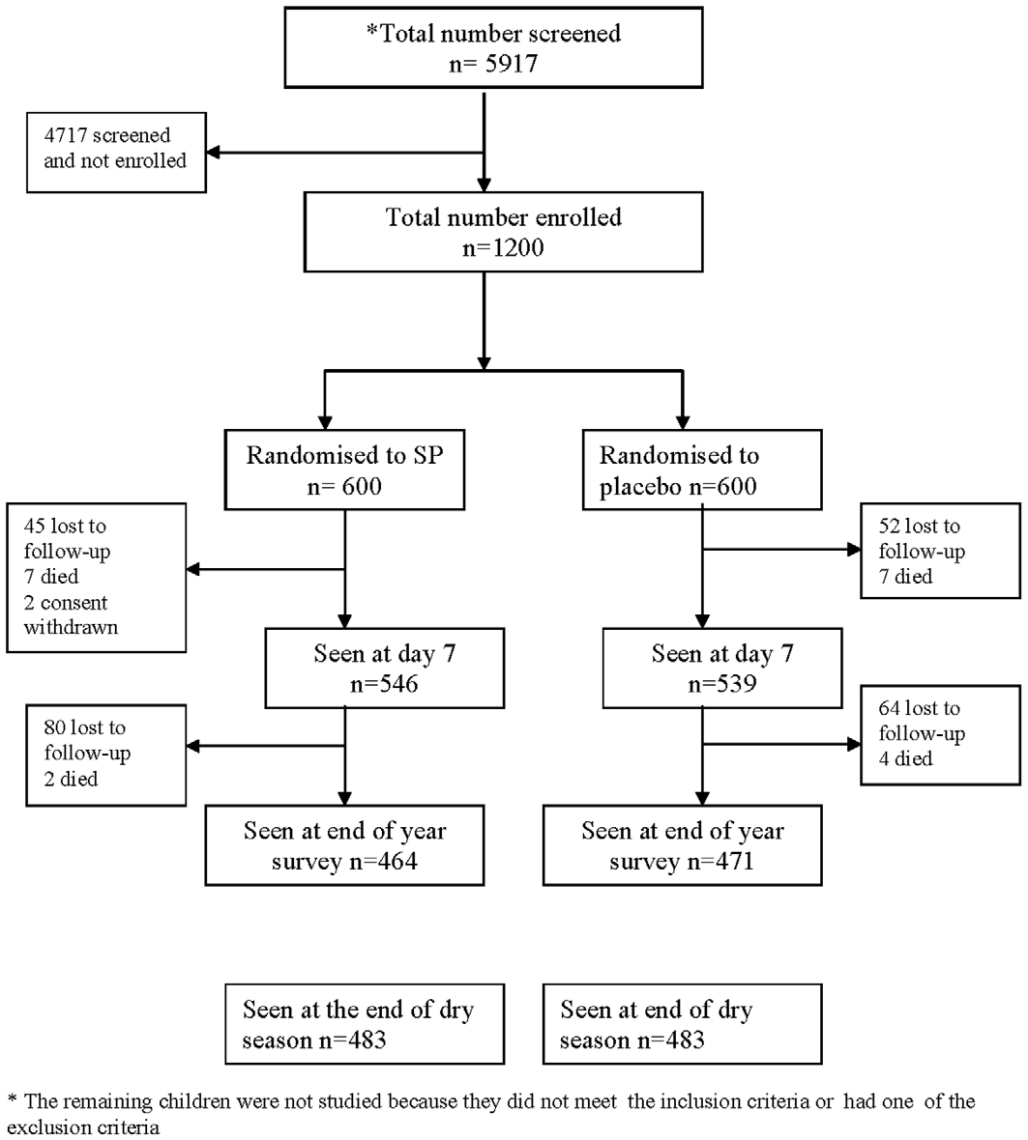
Trial profile.

During the two-year study period, 5917 children with pallor who presented at the five trial sites were screened. 1200 eligible children were enrolled (600 during each year of the study, Figure 2). The remaining children were not enrolled because they did not meet the inclusion criteria or had one of the exclusion criteria. Recruitment started in August during the first year of the study and in July during the second year, but the majority of study subjects (80%) were recruited during the peak period for malaria transmission (September, October and November) and thus were eligible to receive only 2 or 3 treatments with SP. 546/600 (91.0%) and 539/600 (89.8%) of the children enrolled into the SP and placebo groups on admission to hospital received their first dose of trial medication. Among the 54 children in the SP group who were not seen at the day 7 visit, 45 were lost to follow-up, 7 died and 2 subjects were withdrawn by their families. In the placebo group, 61 did not come for the day 7 follow-up and of these 52 were lost to follow-up, 7 died and 2 were withdrawn by their families. 951/1200 (79%) subjects were seen at the end of the malaria transmission season, of these 942 had received at least one dose of IPT or placebo. 966/1200 (80%) subjects were seen at the end of the following dry season.

### Baseline characteristics of the study children

Baseline characteristics of enrolled patients were similar for the two treatment groups (Table 1). The prevalence of malaria parasitaemia in the SP and placebo groups were 62% and 61% respectively and mean parasite densities were similar in each group (Table 1). In addition, there were more children in the placebo group who had splenomegaly compared to the SP group. More children in the SP group slept under a bednet compared to children in the placebo group when bednets were inspected at the end of the transmission season.

**Table 1:** Admission characteristics of children in the two study groups.

Variable	SP	Placebo
	**N = 597	**N = 598
Age (months)		
(mean, SD)	28.4 (18)	28.7 (19)
Sex (male) %	52.3%	52.1%
Ethnic group		
Mandingo	228(39%)	227(38%)
Wollof	74 (13%)	91 (15%)
Fula	98 (17%)	96 (16%)
Jola	85 (14%)	88 (15%)
Others	102 (17%)	93 (16%)
Recruitment centre		
RVTH	160 (27%)	166 (28%)
MRC	117 (20%)	114 (19%)
Brikama	155 (26%)	152 (26%)
Essau	124 (21%)	126 (21%)
Sibanor*	36 (6%)	36 (6%)
Previous chloroquine treatment (%)	62.4	60.6
Educational attainment of mother (years in school), (mean, SD)	2.1 (5.7)	2.0 (3.6)
Resp rate/min (mean, SD)	40.0 (13)	39.3 (12)
Mean haemoglobin g/dl (SD)	5.1 (1.2)	5.2 (.053)
Temp °C (mean, SD)	37.5 (0.91)	37.56 (.88)
Pulse rate/min (mean, SD)	121 (21)	120 (21)
Splenomegaly (%)	19.8	24.7
Hepatomegaly (%)	26.7	27.2
Proportion with malaria parasitaemia	62%	61%
Geometric mean density (95% CI) IQR	17710 (136223, 23024) (0, 51000)	15296 (11668, 20050) (0, 47600)
Sleeps nightly under intact or impregnated net#	20.7	15.3

*Recruitment only during year 2.

**Data missing for 2 and 3 subjects in the placebo and SP groups respectively.

#bednet use was determined at the end of the transmission season.

### Mortality, morbidity and clinical course in hospital

Fourteen of the 1200 children enrolled died in hospital before receiving their first dose of trial medication (7 in each group). Two had pneumonia, one developed cerebral malaria, and 11 died of severe anaemia. Nine children died despite having received a blood transfusion. At the day 7 follow-up, the prevalence of malaria parasitaemia in the SP and placebo groups was 29/546 (5.3%) and 25/539 (4.6%) respectively. These patients were asymptomatic and most had only low-level parasitaemia. Mean haemoglobin (SD) concentrations 7 days after discharge were also similar in the two treatment groups [7.51 (3.6) g/dL and 7.45(3.3) g/dL]. 12.5% and 13% of the children in the SP and placebo groups had haemoglobin concentration of less than 5g/dl at the day 7 follow-up visit.

### Compliance with IPT

Because administration of IPT was terminated at the end of the malaria transmission season, the number of times that a child should have received SP or placebo varied from 1 to 6 (Table 2). Compliance was initially high but decreased progressively in children who were required to take the most doses.

**Table 2:** Number of doses of trial medication received and compliance (percentage).

Dose number	SP			Placebo		
	Number of children scheduled to receive the dose	Number of children that received the dose	*Percentage Compliance	Number of children scheduled to receive the dose	Number of children that received the dose	*Percentage Compliance
1	600	546	91%	600	539	90%
2	520	409	79%	514	410	80%
3	327	222	68%	321	224	70%
4	173	101	58%	168	99	59%
5	71	27	38%	64	29	45%
6	9	2	22%	9	3	33%

### Overall impact of IPT on mortality, morbidity and nutritional status

Between the time of first administration of IPT and the end of the malaria transmission season, six children died (2 in the SP group and 4 in the control group). In the SP group, one child died of severe anaemia and another died of severe malaria. In the placebo group, four children died of severe malaria. Nine children died during the dry season following the period in which IPT was given, 4 in the SP group and 5 in the placebo group. In the SP group, two children died of severe anaemia and one each of malnutrition and acute respiratory infection. In the placebo group, 2 children died of severe malnutrition and one each of HIV infection, a road traffic accident and severe anaemia. Thus, overall there were 6 deaths in children who received SP and 9 in those who received placebo.

Twelve study children were re-admitted to one of the health facilities in the study area during the follow-up period; 5 and 7 in the SP and placebo groups respectively. Three children were admitted because of severe anaemia; one in the SP and two in the placebo group. One of the children in the placebo group was admitted on two occasions because of severe malaria anaemia. The protective efficacies of the intervention against various morbidity endpoints detected during outpatient or dispensary visits are shown in Table 3. There were 257 visits to the OPD in the placebo group and 184 in the SP group, a reduction of 29% (95%CI 13%,42%). The intervention provided 43% (20%,59%) efficacy against episodes of documented fever (axillary temperature of ≥37.5°C). Protective efficacy against upper respiratory infection, skin/soft tissue infection and gastroenteritis was 21%, 17% and 41% respectively. However, the numbers of these events were small and corresponding 95% confidence intervals are wide.

**Table 3:** Morbidity during the malaria transmission period in children who received at least one dose of SP or placebo.

Outcome	SP			Placebo			Protective efficacy# (95% CI)	P value
	Events	Person months at risk	Incidence rate*/100 person months	Events	Person months at risk	Incidence rate*/100 person months		
Total outpatient visits	184	1288.2	14	257	1279.4	20	29% (13%,42%)	0.001
OPD visits with fever (temp ≥37.5°C)	59	1288.2	4.6	102	1279.4	8.0	43% (20%,59%)	0.001
Anaemia (Hb<7g/dL)	19	1288.2	1.5	24	1279.4	1.9	21% (−49%,58%)	0.466
Anaemia (Hb<5g/dL)	2	1288.2	0.16	9	1279.4	0.70	78% (−3%,95%)	0.055
Upper respiratory tract infection	50	1288.2	3.9	63	1279.4	4.9	21% (−18%,47%)	0.248
Skin/soft tissue infection	25	1288.2	1.9	30	1279.4	2.3	17% (−55%,56%)	0.558
Gastroenteritis	9	1288.2	0.70	15	1279.4	1.2	41% (−33%,74%)	0.207

*Incidence from the date of first dose of IPT with SP or placebo up to the end of December the same year.

#estimated as 100×(1-HR) where HR is the hazard ratio from Cox regression, without covariate adjustment. Efficacy against anaemia Hb<7, adjusted for effects of bednet use, centre, baseline haemoglobin concentration and presence of parasitaemia at baseline was 8.2% (95%CI −81%,53%), compared with an unadjusted estimate among the subset of individuals with non-missing covariate data of 13% (−69%,56%).

Children who had received IPT with SP were better nourished at the end of the malaria transmission season than children who had received placebo. Weight-for-height and mid upper arm circumference were significantly higher in the SP than in the placebo groups at the December cross-sectional survey (Table 4).

**Table 4:** Anthropometric findings at the end of the malaria transmission season.

Anthropometric measurement (mean, SD)	SP	Placebo	P value
Height-for-age (z score) Mean (SD)	−0.51 (1.1)	−0.59 (1.4)	0.31
Weight-for-height (z score) Mean (SD)	−0.87 (1.2)	−1.03 (1.3)	**0.04**
Weight-for-age (z score) Mean (SD)	−0.86 (1.5)	−0.93 (1.4)	0.32
Mid upper arm circumference (cm) Mean (SD)	14.91 (1.9)	14.67 (1.5)	**0.02**
Stunted	10.9%	18.5%	**0.02**
Underweight	22.8%	24.4%	0.58
Wasted	17.7%	20.7%	0.28

### Protective efficacy of IPT against anaemia

The proportions of children with a Hb concentration of <7 g/dL at the end of the malaria transmission season, the primary trial endpoint, were similar in the two groups of children, 14/464 (3.0%, 95%CI 1.5%, 4.6%) in children who received SP and 16/471 (3.4%, 95%CI 1.8%, 5.0%) in those who received placebo (prevalence ratio 0.89 (0.44,1.8) P = 0.742). Only one child, in the placebo group, had a Hb concentration of <5g/dL. The mean haemoglobin (SD) concentration at the end of the malaria transmission season was slightly higher in children in the SP group [10.8 (1.7) g/dL ] compared to the placebo group [10.6 (1.8)g/dL ], difference 0.23 (0.002,0.45) P = 0.048 (adjusted for baseline Hb and other covariates) but this difference is unlikely to have been clinically significant. The cumulative number of children who had anaemia (Hb<7g/dL) at OPD visits during surveillance or at the end of the transmission season was 32/470 (6.8%) in the SP group and 42/477 (8.8%) in the placebo group (ratio 0.77 (0.50,1.2) P = 0.252), and of these 4/465 (0.9%) had Hb<5 in the SP group compared to 11/462 (2.3%) in the placebo group (ratio 0.37 (0.12,1.2) P = 0.074). Similar results were obtained after adjusting for covariates (Table 3); there was no evidence of an interaction between treatment group and bednet use.

When the subgroup who had received blood transfusion were considered, the prevalence of moderate to severe anaemia (Hb<7 g/dL) at the end of the transmission season was slightly higher among children who had been transfused (5/43 (14.7%) in the placebo group compared to 2/30 (6.67%) in the SP group) but there was no evidence of interaction between intervention group and having been transfused (P = 0.350).

### Protective efficacy of IPT against malaria

**Figure 3:**
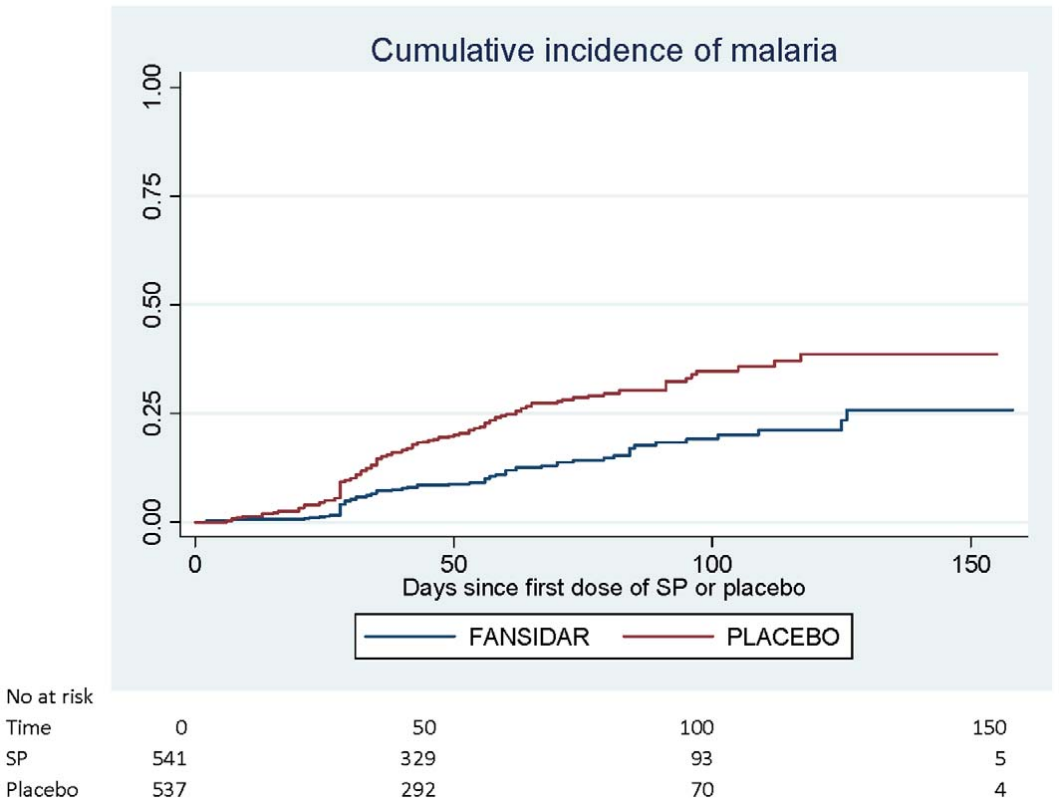
Kaplan Meier estimates of the cumulative incidence of malaria.

During the malaria transmission season following enrolment, there were 30 episodes of malaria with high parasitaemia (>5000/µl) in children who received SP compared to 68 in those who received placebo, a protective efficacy of 56% (30%,73%). When all episodes of malaria are considered, 91 episodes of clinical malaria were recorded in children who received SP compared with 192 in children who received placebo giving an efficacy of 53% (37%,65%)[Table 5]. There was no evidence of an interaction between treatment group and bednet use. Figure 3 shows the timing of first episodes of malaria in each group (logrank test: P<0.001).

**Table 5:** Incidence of malaria during the malaria transmission period among children who received at least one dose of IPT.

Outcome	SP			Placebo			Protective efficacy# (95% CI)	P value
	Events	Person months at risk	Incidence rate*/100 person months	Events	Person months at risk	Incidence rate*/100 person months		
All episodes with >5000 parasites per µL	30	1288.2	2.3	68	1279.4	5.3	56% (30%,73%)	0.001
All episodes of clinical malaria	91	1288.2	7.1	192	1279.4	15	53% (37%,65%)	<0.001
First or only episode with >5000 parasites per µL	26	1244.9	2.1	60	1184.6	5.1	58% (33%,73%)	<0.001
First or only episode of clinical malaria	70	1170.0	6.0	133	1054.4	13	52% (36%,64%)	<0.001

#estimated as 100×(1-HR) where HR is the hazard ratio from Cox regression, without covariate adjustment. Efficacy against the first episode of malaria, adjusted for effects of bednet use, centre and age at enrolment was 54% (37%,66%), compared with an unadjusted estimate among the subset of individuals with non-missing covariate data of 51% (33%,64%). The corresponding estimates for the first episode of malaria >5000/uL were 58% (31%,74%), compared with 56% (28%,73%) unadjusted; for all episode of malaria, 52% (38%,63%) compared to 50% (33%,63%) unadjusted; and for all episodes of malaria >5000/uL, 58% (32%,74%), compared to 55% (27%,73%) unadjusted.

Seventy-two of 460 children (16%) in the SP group had asexual stage *P.falciparum* parasitaemia compared with 119/473 (25%) in the control group (prevalence ratio 0.62 (0.48,0.81) P<0.001) but very few children in either group had a parasitaemia of ≥5000/µL (6 (1.3%) SP, 16 (3.4%) placebo, prevalence ratio 0.39 (95%CI 0.15,0.98) P = 0.037). There was a statistically significant difference in the prevalence of splenomegaly between the two groups at this time point but the prevalence of splenomegaly was low in both groups (2.9% in SP vs 6.5% in placebo group)( p = 0.006).

Interviews with mothers suggested that 46 and 50 children in the SP and placebo groups respectively received antimalarial treatment during the dry season.

### The effect of the intervention on parasite genotypic markers of resistance to SP

One hundred and ninety-five (75%) and 182 (70%) of 259 first positive samples collected at enrolment were successfully analysed for polymorphisms in *dhfr* (codons 51, 59, and 108) and *dhps* (codon 437 and 540) genes respectively. The numbers genotyped among 78 samples collected from parasitaemic children during the December cross-sectional survey were 58 (74%) and 44 (56%) for the two genes respectively. The remainder were not analysed because there were no detectable PCR products. The prevalence of triple mutations in the *pfdhfr* gene associated with pyrimethamine resistance (codons 51, 59 and 108) increased during the period of surveillance in children who received monthly SP, from 87% (72/83) at the time of clinical presentation to 100% (14/14) at the end of season, and in those who received placebo from 82% (92/112) at the time of clinical presentation to 95.5% (42/44) at the end of season. Neither of these increases were statistically significant (p = 0.32 and p = 0.06 respectively). The prevalence of the codon 437 mutation in the *pfdhps* gene associated with resistance to sulphonamides did not change significantly during the study period in either arm of the study. For the SP treatment group, the prevalence was 49% (39/79) at the time of enrolment and 21% (3/14) in December (p = 0.1), and in the placebo group the prevalence was 48.5% (50/103) at the enrolment and 37% (11/30 (37%) in December (p = 0.35). The 540E allele in the *dhps* gene associated with sulphonamide resistance was not present in the study population.

### Safety and tolerability

No severe skin reactions suggestive of the Stevens Johnson syndrome were seen. Minor symptoms recorded during the 30 days after the administration of each treatment were similar in the SP and placebo groups (data not shown). No child was withdrawn from the study because of an adverse event due to SP.

## Discussion

Intermittent preventive treatment (IPT) was developed first as a strategy to control malaria in pregnancy and then adapted to malaria control in infants and older children. We have further extended this approach to anaemic children because this group represent a vulnerable group who could benefit from a highly effective malaria control strategy.

We did not find that monthly treatment with SP had any significant impact on the prevalence of moderate or severe anaemia in previously anaemic children followed through a malaria transmission season, the primary endpoint for this trial. Less than 4% of children in either the intervention or the control group had a Hb concentration of less than 7 g/dL when seen at the end of the malaria transmission season. Children in the intervention group did have a higher mean haemoglobin concentration than the control children (10.8g/dL vs 10.6g/dL), a difference that is unlikely to be clinically important. Thus, in The Gambia, an area of moderate malaria endemicity and highly seasonal transmission, provision of oral iron supplementation for a four-week period and ready access to a health facility seem adequate to prevent previously anaemic children from relapsing. In our study, iron supplementation was given for only one month, a decision based in part on the result of a previous pilot trial carried out at RVTH, Banjul which showed that the mean PCV was significantly higher in children with severe anaemia who received one month of iron supplementation than in those treated by blood transfusion alone and that by the end of the malaria transmission season a majority of the children treated with iron had normal haemoglobin concentrations [5]. In an study undertaken in young children with anaemia in Tanzania, it was shown that children who received iron supplementation and monthly SP for three months had a lower prevalence of anaemia than those who received these interventions for only a month but the difference between groups was modest and interpretation of the results of this trial is complicated by differences in the duration of follow-up [23]. Sustaining compliance with iron supplementation for three months would be difficult and there are concerns that iron supplementation may increase the risk of malaria in highly endemic areas [24].

The overall prevalence of moderate anaemia at the end of the malaria transmission season in children previously treated for moderate or severe malaria was much less than we had anticipated based on previous experience in The Gambia. This may be due to the fact that the incidence of malaria in The Gambia began to fall at about the time that this study was conducted [25] so that malaria has become a much less important cause of anaemia than was the case 10 or more years ago when earlier studies were done. If this is the case then SP would not be expected to have a significant effect on the prevalence of anaemia although remaining partially protective against malaria.

We did not observe the high rate of recurrence of anaemia and the associated mortality observed in previous studies in Kenyan when children with severe anaemia were followed up following their discharge from hospital [11], [12]. The mortality rate during a period of approximately 6 months following discharge from hospital was 1.1% in the SP group and 1.7% in the placebo group. This difference might be due in part to differences in enrolment criteria. In our study, children with moderate or severe anaemia (Hb<7g/dL were enrolled whilst the Kenyan trials enrolled only children with severe anaemia (Hb<5g/dl). Severe anaemia is associated with an increased risk of death [26] but there is less evidence of increased mortality associated with moderate anaemia [26], [27]. Another important difference between the two study areas is the pattern of malaria transmission. In The Gambia, malaria is seasonal and most cases of anaemia occur towards the end of the malaria transmission season so that much of the follow-up period occurred during the period when very little malaria transmission was taking place. In contrast, malaria transmission is higher and more perennial in the area of Kenya where the previous observations were made. In The Gambia, the majority of the children with severe anaemia were treated with quinine and SP. Both these drugs remain effective and eliminated parasitaemia in nearly all cases. Studies in Kenya have shown that persistent parasitaemia at the time of discharge and during follow-up due to only partially effective antimalarial treatment are a strong predictor of post-discharge mortality [11], [12], [28]. All the children enrolled in the Gambian study had easy access to health care during the study period and this could also have contributed to the low mortality observed.

Despite its lack of a significant impact on anaemia, IPT with SP was partially effective at protecting against clinical attacks of malaria with a protective efficacy of 56%. The fact that the protective efficacy was not higher should not be taken as an indicator of SP resistance as efficacy measurements included children who received no drug or who spent several months without drug cover. A formal analysis of day 28 parasite clearance rate following SP treatment would almost certainly have given a higher efficacy figure. Administration of a total of 1700 doses of SP to 546 children (on an average of 3.1 occasions per child) prevented 101 episodes of malaria during an average period of follow up of 2.7 months, a saving of 1 clinical episode of malaria for every 17 doses of SP given, a figure comparable to that seen in studies of IPTi. The reduction in the number of clinical episodes of malaria was associated with a 27% reduction in the overall number of clinic visits during the follow-up period and children who received SP were better nourished than those who received placebo, consistent with findings in other studies of IPT in children [29]. Deaths and hospital admissions were fewer in children who received SP than in those who received placebo but numbers of events are too small for conclusions to be drawn.

The level of protection against clinical malaria episode observed in this study is similar to that observed in two other previous studies of IPT in Kenyan children with mild to moderate anaemia [30], [31]. In the first study, conducted in 328 afebrile children aged 2–36 months who had a haemoglobin concentration of between 60 and 110 g/dl, administration of SP and SP plus iron decreased the number of attacks of clinical malaria (hazard ratios of 0.59 and 0.76 respectively) but the number of episodes of malaria was small and this reduction was not statistically significant [31]. In a second trial conducted in 546 children aged 2–36 months who had a haemoglobin concentration of 70–110 g/dL were enrolled. Clinical attacks of malaria were again reduced by about a half in children who received SP alone (hazard ratio of 0.47) but the difference from the placebo group was not statistically significant (p = 0.07) [30]. Thus, these two trials showed similar point estimates of levels of protection with chemoprevention with SP to those observed in the Gambian study although the confidence intervals were wide.

The possibility that large-scale drug administration on a regular basis will enhance the spread of drug resistance is a concern for malaria control by chemoprophylaxis or IPT but our study was not powered to address this issue, and so our results on the prevalence of markers of resistance must be interpreted with caution. Among the samples examined on enrolment there was a high prevalence of the triple mutation allele at codons 108, 51 and 59 in the *dhfr* gene associated with pyrimethamine resistance in both the SP and control groups, and these frequencies increased during the malaria transmission season in both groups. The 437G allele in the *pfdhps* gene associated with resistance to sulphonamides did not increase in frequency, and the 540E allele in the *dhps* gene was not present in this population. We cannot ascertain whether the increased in molecular markers of resistance to pyrimethamine was related to the intervention or an indication of a more general process taking place in the study area.

Compliance was high among study subjects who were scheduled to take three or less doses of the trial medication. However, for study subjects who were scheduled to take more than three doses, compliance was initially high but decreased progressively with a tendency for this effect to be more marked in children in the SP group. However, overall there was no significant difference in compliance observed between the SP and placebo groups suggesting that SP did not induce any adverse effects that were of concern to the mother or guardian. During the study, treatment was available to study subjects free of charge and parents were provided with transport money so lack of funds was not the cause of the low compliance associated with increased doses taken. Distance from a health facility was also not a likely cause as most of the study area is urban/peri-urban with relatively good access to health facilities. It is more likely that the decrease in compliance seen with increased length of follow-up was due to general apathy towards the project. Before an intervention such as this could be implemented widely, issues relating to compliance need to be investigated and an extensive education programme would be needed.

SP was well tolerated and no severe skin reactions or other treatment related adverse event was reported. These finding are encouraging and are similar to those previously reported on the safety of SP in African children when used for IPTi [32] or IPTc.

Weaknesses of the study include the fact that majority of the study children received their treatment towards the end of the season so these children received only one or two doses of intermittent treatment decreasing our ability to discriminate between groups. In addition, a recent pharmacokinetic study suggests that current dosing of sulfadoxine-pyrimethamine in young children is inadequate and that children aged 2–5 years should be treated with 1 g sulphadoxine and 50 mg pyrimethamine to achieve drug concentrations equivalent to those needed in adults for treatment of uncomplicated malaria [33]. This could have resulted in some study children receiving lower than recommended doses of SP reducing the protective efficacy of IPTc.

In The Gambia, intermittent administration of SP to children previously admitted to hospital with anaemia did not have a clinically important impact on their haemoglobin concentration by the end of the transmission season although it did protect against malaria. Different results may be obtained in areas where transmission of malaria is higher and perennial.

## Supporting Information

Protocol S1 (0.11 MB DOC)

Checklist S1 CONSORT checklist.
(0.05 MB DOC)
