# Supplementary material for: Prevention of the Recurrence of Anaemia in Gambian Children Following Discharge from Hospital
Source: PLoS One. 2010 Jun 21;5(6):e11227. doi: 10.1371/journal.pone.0011227 (PMC2888645; doi:10.1371/journal.pone.0011227)
Supplement: Protocol S1 — (0.11 MB DOC) [file pone.0011227.s001.doc]

**TITLE OF PROPOSAL**

Chemoprevention with sulfadoxine-pyrimethamine to prevent morbidity in Gambian children treated for severe anaemia

**SUMMARY**

Severe anaemia is a frequent cause of admission to hospitals in tropical Africa and about 10% of such children die. In endemic countries, anaemia has multiple causes such as nutritional deficiencies, infections and haemoglobinopathies. However, *Plasmodium falciparum* infection is believed to be the major contributory factor to the aetiology of severe anaemia. Severe anaemia is usually treated by blood transfusion although transfusion carries the attendant risk of transmission of HIV and other blood-borne infections. Thus, there is a need to explore novel strategies to reduce the incidence of severe anaemia in high-risk groups such as children with suboptimal haemoglobin levels because these children are at increased risk of developing severe anaemia if they develop a malaria infection before their haemoglobin level has normalized. Therefore, it is proposed to study whether monthly chemoprevention with sulphadoxine/pyrimethsmine (S/P) given during malaria transmission season can protect Gambian children from developing severe anaemia. After receiving treatment from the hospital, 1200 Gambian children with a haemoglobin concentration of less than 7 g/dL will be randomised to receive either monthly SP or placebo during the rest of the malaria transmission season. Morbidity will be monitored throughout the rainy season. Study subjects will be seen at the end of the dry season to document morbidity and mortality.

The primary endpoint of the trial will be the proportion of children with haemoglobin concentration of less than 7g/dL at the end of the malaria transmission season. An important secondary end-point will be the number of OPD attendances with malaria at the end of the malaria transmission season.

**RESEARCH PROPOSAL**

**1.0 Background to the study**

Anaemia is an important public health problem in sub-Saharan Africa with major social and economic implications. Anaemia causes decreased exercise tolerance and intellectual performance and leads to impairment of normal development. In endemic countries, the prevalence of anaemia in the community is estimated to be 49-89% and that of severe anaemia 1.3-6.4% ^1-6^. The prevalence of severe anaemia in hospitalised patients is estimated to be in the range of 7-29%^7-12^. In malaria endemic countries, the incidence of severe anaemia and age specific rates of anaemia correlate strongly with the intensity of *Plasmodium falciparum* malaria transmission, and significant haematological recovery has been seen after malaria control trials^13,14^. In endemic countries, anaemia has multiple causes such as nutritional deficiencies, infections and haemoglobinopathies. However, *P. falciparum* infection is believed to be a major contributory factor to the aetiology of severe anaemia**.**  Though severe anaemia is an important complication of falciparum malaria, it has not received as much attention from the research community in recent years as cerebral malaria. In areas of seasonal transmission, cerebral malaria is the dominant form of severe malaria^15^. In contrast, in areas of high transmission, severe anaemia in young children is the most prevalent form of severe malaria^16^. Although mortality from severe anaemia, about 10%, is less than that from cerebral malaria (20%-30%), it is likely that several hundred thousand deaths occur from this condition each year.

Prevention of the development of anaemia in children who experience malaria infection depends on both effective parasite clearance and enhancement of the bone marrow response ^17-19^. Thus, effective antimalarial drugs should reduce parasite persistence and quicken haematological recovery. Despite increasing evidence of widespread resistance and the coincidental increase in prevalence in severe malaria anaemia, chloroquine remains the officially recommended treatment for uncomplicated malaria in The Gambia and many other African countries. Resistance of *P. falciparum* to chloroquine was first detected in The Gambia in 1986, much later than in East Africa, but resistance has subsequently become increasingly prevalent^20^. A national survey undertaken in 1987 showed an overall prevalence of R1/R2 resistance of 5%: the highest prevalence (16%) was found in the central part of the country, the lowest (<1%) in the Western Region where chloroquine was most widely available^21^. However, by 1992 the prevalence of R1/R2 resistance among children seen at MRC Fajara had increased to 51%^22^ and the following year a 65% level of R1/R2 resistance was found among children who presented to the health centre at Farafenni^23^. In both studies, children treated with chloroquine had lower PCV’s than those treated with S/P one month after presentation. In a more recent study undertaken in 1998, a R1/R11 resistance level of 30% was found in Farafenni^24^.

Blood transfusion remains the mainstay of treatment for severe anaemia. However, providing a safe and reliable blood transfusion service is a very challenging task in many resource poor health facilities in sub-Saharan Africa and the rapid spread of HIV infection means that the risk of transfusion is substantial^25^. Severe anaemia is often unrecognised because the signs and symptoms of anaemia are non-specific and it is likely that many such children die in the community before they can receive appropriate treatment. Thus, in the absence of an affordable, safe and effective vaccine, there is an urgent need for new inexpensive strategies that will prevent the development of severe anaemia especially in high-risk groups such as children with suboptimal haemoglobin levels. These children are likely to be at increased risk of developing severe anaemia if they experience malaria infection before their haemoglobin has returned to the normal range. Such strategies include intermittent preventive treatment with an effective antimalarial, insecticide treated bed nets (ITNs) and iron supplementation.

### Chemoprophylaxis and intermittent preventive treatment

Antimalarial chemoprophylaxis in endemic countries has been shown to reduce malarial morbidity, school absenteeism, and all cause mortality but it is no longer advocated because of concerns over its possible effect on the development of resistance, logistics constrains and its potential effect on the development of natural immunity. Chemoprophylaxis can lead to loss of acquired immunity or delay its development and this can potentially cause an increase in episodes of clinical malaria when the intervention is withdrawn. To take advantage of the protective effects of chemoprophylaxis whilst reducing its possible adverse effect on the development of natural immunity to malaria, intermittent preventive treatment (IPT) with an effective an antimalarial has been advocated. IPT involves giving a full treatment dose of an antimalarial drug at specific times, regardless of the presence or absence of malaria parasites. Since treatment is only given on an intermittent basis, this intervention is less likely to interfere with the development of immune responses than sustained chemoprophylaxis. The results of trials conducted using IPT have so far been encouraging.

To determine whether children who had experienced an attack of severe malarial anaemia might benefit from a limited period of chemoprophylaxis, we undertook a pilot trial in 1994 in which Maloprim^R^ (pryrimethamine/dapsone) chemoprophylaxis was given after children had been discharged from hospital. Children who received chemoprophylaxis had fewer episodes of malaria. However, there was no significant difference in the mean haematocrit between those who received chemoprophylaxis and the control group at the end of the follow-up. This was most likely due to the fact that chemoprophylaxis was only started after the end of main malaria transmission season when there was hardly any transmission of malaria taking place^26^

# Insecticide treated nets

Insecticide treated-mosquito Nets (ITNs) are a key malaria control tool. Several trials have shown ITNs to be a promising approach to malaria control in areas of Africa, Asia and the Pacifics. A recent Cochrane reviewe^27^ concluded that ITNs reduce overall mortality by about 20% in Africa (range 14%-29%^28-30^) and clinical attacks of malaria by 50%. Overall, mean haematocrit was 1.4% higher among children sleeping under ITNs compared to those without nets. A study in Tanzania showed that ITNs had a protective efficacy of 62% against anaemia^31^.

## Iron supplementation for the prevention anaemia

It has been shown that a considerable number of children with severe malaria are iron deficient^32^. Several studies have attempted to evaluate the risk benefit of iron supplementation in malaria-endemic areas. Some studies reported that iron supplementation increased the risk of acquiring or reactivating malaria, while others reported no significant adverse effects. Difference in malaria endemicity, trial design, endpoints and dosage regime may have accounted for these contradictory results^33-43^ . Recently, a trial in an area of intense malaria transmission in Southern Tanzania assessed the impact of iron supplementation (2mg/kg given daily between 2 and 6 months of age), malaria chemoprophylaxis (pyrimethamine /dapsone), both or neither, on the prevention of severe anaemia in infants^44^. Iron supplementation had a protective efficacy of 28.8% (95% CI 6.3 –45.8%) against the development of anaemia and it did not increase the risk of malaria. In another more recent study in an area of seasonal transmission intermittent treatment with S/P plus iron or S/P alone reduced the incidence of severe malarial anaemia and clinical attacks of malaria^48^.

**2.0 Objectives**

**2.1 Goal:**

To contribute to the development of evidence based strategies for preventing morbidity / mortality associated with malaria and anaemia.

**Specific objectives:**

1. To assess the effectiveness of monthly with S/P on haematological recovery in children who have been treated for severe anaemia.
2. To measure compliance with the intervention
3. To investigate whether or not infection with chloroquine and S/P resistant strains contributes to the pathogenesis of severe anaemia

3.0 Study design and methods

3.1 Study site:

The study will be based at the 75-bed paediatric unit of The Royal Victoria Hospital (RVH), which is located in the capital, Banjul. This is the main referral hospital for the country. The paediatric unit admits about 7000 children a year, of whom about 800 are anaemic (PCV < 33%). About 250 children with severe anaemia are transfused each year. Children seen at the MRC Hospital will also be eligible for enrolment in the study. Among Gambian children, malaria is the most important cause of anaemia.

### **3.2 Malaria in The Gambia**

Transmission of malaria in The Gambia is seasonal. The climate is typical of sub-Sahelian Africa, with a long dry season which lasts from November-June, followed by a relatively short rainy season from July-October. Rainfall averages about 600 mm per year. Morbidity and mortality from malaria follow this pattern, both occurring more frequently during the rainy season, with a peak in the month of October. *P. falciparum* is the dominant species, being responsible for all severe diseases and for over 95% of clinical attacks. A few cases of clinical malaria are caused by *P. malariae;* *P. ovale* is seen rarely. In rural areas, children experience 0.5-1.0 clinical attacks of malaria a year. Over 90% of clinical attacks occur during the rainy season. Parasite and spleen rates in children aged 1-4 years range from 25% -50%. Acute respiratory infection and malaria are the most common causes of death in children who survive the first month of life. Malaria causes about 4% of deaths in infants and 25% of deaths in children 1- 4 years of age.

Chloroquine is still the officially recommended first line treatment for uncomplicated malaria, but sulphadoxine/pyrimethamine is used widely and quinine is used for the treatment of severe malaria. Anti-malarials can be purchased in several pharmacies in Greater Banjul. Bed nets are used widely in rural areas and The Gambia Government makes permethrin available at a modest cost at the beginning of the malaria transmission season.

**3.4 Enrolment of children**

Children will be enrolled from the paediatric unit of the Royal Victoria Teaching Hospital, Banjul, the MRC Hospital, Fajara, Brikama Health Centre or Essau Health Centre during the first year of the study. The study will be extended to WEC clinic in Sibanor during the second year . Study subjects will be enrolled by non-coercive methods if they met the inclusion criteria and had none of the exclusion criteria.

Inclusion criteria:

1. Age: 3 months to 9 years.
2. Haemoglobin less than 7 g/dL
3. Residence within 30 km of the study centre and availability for the duration of the study period.
4. Informed consent to participate in the study given by the parent or guardian.

## Exclusion criteria:

1. Simultaneous participation in any other trial.
2. Allergy to sulpha drugs.
3. Residence > 30 km from the recruitment site.
4. Lack of consent.

3.5 Screening process and enrolment

All children in the right age group who present to the ward/OPD will have their haemoglobin concentration measured and blood film examined for the presence of malaria parasite. Children with a haemoglobin concentration of less than 7 g/dL will be offered assessment by a project doctor. If assessment is permitted, and the patient fulfils the entry criteria, then written consent will be sought from the parent/guardian. A screening record will be maintained to log all patients who are considered as potential patients but who are excluded. The study will be explained in the parents/ guardians’ preferred language, and written informed consent will be obtained on approved forms.

On entry into the trial a detailed clinical history, drug history, family history and information on bednet usage and impregnation will be obtained. The study physician will examine the child, document nutritional status and record the findings in a case report form. In addition, a detailed address will be obtained. Each study child will be provided with a photo ID card to facilitate identification at each contact.

A 5-ml baseline blood sample will be obtained from the first 142 patients seen at the Royal Victoria Teaching Hospital or MRC Clinic for determination of the following:

- Hb, red cell indices, white cell and platelet count (Coulter counter)
- Reticulocyte count
- Markers of iron status (serum iron and ferritin) (first 100 children)
- Sickling test and, if positive, haemoglobin electrophoresis
- Stool examination for hookworm and other parasites (first 100 children)

Other investigations such as chest x-ray and urine examinations will be carried out as clinically indicated. Whenever possible the cause of the anaemia will be determined

3.6 Inpatient management

Children will be treated with parental quinine and given a standard dose of SP on discharge from hospital. In the case of children with impaired consciousness, lumbar puncture will be carried out to exclude concomitant meningitis. Children with a haemoglobin concentration of less than 5 g/dL and/or signs of respiratory distress will be scheduled for immediate transfusion with whole blood in accordance with national guidelines. All patients will received iron for 28 days , treatment starting at the time of their discharge from hospital when their malaria had been treated. Patients will be treated for other conditions as clinically indicated. Children will be kept in hospital until all signs of respiratory distress has subsided and until Hb measurements show an increase in Hb compared to the admission Hb. Patients will be asked to return for follow-up 7 days after admission. Before discharge from hospital, each patient will be provided with a photo ID card and the address of the patient obtained.

3.7 Treatment regimens

Treatment will be given as follows:

S/P (tablets containing 500 mg sulfadoxine/ 25 mg pyrimethamine) at an approximate dose of 1.25 mg pyrimethamine/25 mg sulphadoxine per kg stat dose.

IM Quinine: 20 mg/kg loading dose, then 10 mg/kg 12 hourly for 5 days.

Iron will be given as syrup at a target dose of 2 mg/kg elemental iron.

3.8 Assessment of resistance to chloroquine and antifolates

To investigate whether or not infection with chloroquine or S/P resistant parasites contributes to the pathogenesis of severe anaemia in Gambian children, filter paper blood samples will be collected from the first 100 children entered into the study who have *P.falciparum* parasitaemia and an equal number of aged matched children who attend the OPD clinic with uncomplicated symptomatic malaria. Nested PCR on extracted DNA will be used to tested for mutation in genes associated with resistance; *Pfcrt* and *Pfmdr* for chloroquine and DHRF / DHPS genes for S/P using standard techniques^45,46^

.

3.9 Discharge from hospital

Study subjects will be individually randomised into either the S/P or the placebo group in a 1:1 fashion at the time of discharge from hospital, although chemoprophylaxis will not be started for another week as the children are likely to have received antimalarials whilst in hospital. Randomisation will be in blocks. Block size will be selected to enhance blinding and maintain balance in the treatment allocation ratio as recruitment progresses

3.10 Home visit

As soon as possible after discharge from hospital, patients will be visited at home by a project field worker to collect information on social economic and environmental risk factors that may predisposes an individual child to develop severe anaemia. A housing survey will be conducted during this visit to determine possible risk factors for malaria which might act as confounders in determination of the primary trial end-point. Field workers will interview the parents/guardians of study subject to record characteristics about the house that might influence mosquito biting behaviour. These items will include building material, an evaluation of the presence of eaves, impregnation and condition of bed nets. During the visit, parents will be encouraged to protect the study child with a bed net.

3.11 First OPD visit after discharge from hospital

One week after discharge, study subjects enrolled in the study will be asked to return to the OPD clinic for routine assessment. At this visit a fingerpick blood samples will be collected for haemoglobin measurement and thick blood film examination for malaria parasite. The first dose of trial medication will be given during this visit. Any medical condition detected during the visit will be treated.

3.12 Randomisation

Children will be individually randomized into either the SP or the placebo group in a 1:1 ratio, using permuted blocks of 12 generated by computer using the STATA program. Blocks will not split across centres. Tablets (enough for 6 doses) will be packed into envelopes bearing the randomization number. The next envelope in sequence will be assigned to the child at the time of their admission to hospital. The randomisation code will be kept in sealed opaque envelopes by the local safety monitor and the MRC Laboratories accountant who alone will know the code, which associates each treatment group with either S/P or placebo.

3.13 Continuation of chemoprophylaxis in the health centre

Study subjects will receive their monthly chemoprevention at their local health centre under direct supervision by staff of the health centre who will be identified by the health centre and trained to take this important role in the study. At this first OPD attendance, mothers/guardians will receive clear oral instructions as to where to go for the prophylaxis. In addition, written instructions in English and the mother/guardian’s preferred local language will be provided to them. Health centre staff will be provided with a list containing the study number, photographs and randomisation group of each study subject. Study subjects will be observed for 30 minutes after dosing and if vomiting occurs within 30 minutes, the dose will be repeated. For infants and young children, the tablets will be crushed and mixed with water. S/P (tablets containing 500 mg sulfadoxine/ 25 mg pyrimethamine) will be given at an approximate dose of 1.25 mg pyrimethamine/25 mg sulphadoxine per kg. Enrolment of patients will start in July and surveillance will continue until end of malaria transmission season.

# 3.14 Morbidity surveillance during the rainy season

Study subjects will be followed throughout the rainy season. Mothers/guardians will be encouraged to take their child to the RVH OPD clinic or the health centre identified as being closest to their home at any time after discharge if the child becomes unwell. Project staff will be based at each of these health centres, initially to identify children in the trial and to ensure that they are seen, properly investigated and treated promptly by health centre staff. At each visit axillary temperature will be recorded using a digital thermometer. A dipstick for diagnosis of malaria will be used to guide treatment if fever (axillary temperature of ≥ 37.5°C) or history of fever within the previous 48 hours is present. In addition, a thick blood smear for malaria parasites will be collected for subsequent confirmation of the diagnosis. Filter paper sample to test for S/P resistance will be collected from all malaria cases detected during morbidity surveillance. Study subjects with documented fever (axillary temperature of ≥ 37.5°C) or history of recent fever and malaria parasitaemia will be treated with S/P and chloroquine. The treatment of study subjects seen at the health centres will be the carried out by health centre staff in accordance with national guidelines. Study patients needing admission will be referred to RVH paediatric unit or the MRC ward. The Health centre ambulance will be used to transfer study patients referred for admission but if this is not available, the field worker will arrange for the use of MRC transport as soon as possible. Study children admitted to the RVH or MRC hospital will be fully documented.

# 3.15 End of malaria transmission season cross-sectional survey

Children enrolled in the study will be seen at the OPD clinic at the end of malaria transmission season for examination by a study physician and a finger-prick blood sample will be obtained for preparation of thick blood smear and determination of haemoglobin concentration. A standardized questionnaire will be administered to the parents/guardians of the study subject, to collect information regarding illness that had occurred since the last visit, symptoms experienced, use of healthcare facilities and use of medicines. Information on the use of bednets will be collected again at this visit.

# 3.16 Dry season follow-up

At the end of the dry season study subjects will be visited once. During this visit, a questionnaire will be administered to document morbidity and mortality. Children admitted to

hospital with severe anaemia may be at increased of death if they develop another disease before their haemoglobin has returned to the normal range. Therefore it is important to document the morbidity and mortality pattern in these children over a period of several months after their discharge from hospital.

# 3.17 Surveillance for overall and cause specific mortality

Deaths will be investigated using the post-mortem questionnaire techniques and cause of death established wherever possible.

3.18 Study end-points:

Primary end-points

- Proportion of children with haemoglobin concentration of less than 7g/dL at the end of the malaria transmission season.

#### **Secondary end-points:**

- Proportion of children with anaemia (Hb <11g/dL) and those with severe anaemia (Hb <5 g/dL) at the end of the malaria transmission season.
- Mean Hb concentration at the end of the malaria transmission season.
- Number of OPD attendances with malaria
- Number of hospital admissions with malaria during the surveillance period.
- Number of episodes of severe malaria during the surveillance period.
- Deaths (all-cause and malaria-specific) during the surveillance period.
- Adherence to the study regimen: The number of doses of medication received by children in each group as a proportion of the total number of doses that should have been received.

**3.19 Withdrawal Criteria**

Patients will be withdrawn from the study for any one of the following reasons.

- Withdrawal of parental / legal guardian’s consent at any stage.
- Adverse event attributable to the study drug.
- Development of one or more criteria for severe malaria as per WHO definition.
- Development of other severe disease.
- Loss to follow-up.

#### **3.20 Data management and analysis**

All baseline, surveillance and laboratory data will be collected on forms designed for the trial. Field and laboratory staff will be trained to follow the procedures set out in a series of Standard Operating Procedures (SOPs). Key data will be double entered and inconsistencies checked; range checks will be used for standard variables. Primary analyses will be performed on subjects with intent to treat (ITT) basis.

**3.21 Case definitions**

The following case definitions of malaria will be used:

1. Documented fever and /or history of recent fever (within 48 hours) + *P. falciparum* asexual stage parasitaemia ≥5000/μL and symptoms suggestive of acute malaria infection.
2. Documented fever and /or history of recent fever (within 48 hours) + any *P. falciparum* asexual stage parasitaemia and symptoms suggestive of acute malaria infection .
3. Fever is defined as an axillary temperature greater than or equal to 37.5°C.

Severe malaria will be classified using the WHO definitions^47^:

**3.22 Sample size calculations**

Assumptions made in the sample size calculations included –

- An intervention that provides at least 30% protection against moderate anaemia will be a useful public heath tool.
- Based on the number of cases of anaemia cases seen at the RVTH and MRC clinic during previous years, it will be possible to recruit 1200 study subjects during the two-year study period.
- 20% of the children will have a PCV of < 20% (moderate anaemia) at the end of the malaria transmission season.
- The attack rate of clinical malaria in children in the control group will be 0.1 episodes per month (a figure based on previous surveys from The Gambia).
- The drop out rate during follow up will be 15%.

A trial with 1200 children enrolled, will have 80% power to detect a 34% reduction in the prevalence of moderate anaemia (PCV<20%) at the end of the malaria transmission season. With a sample size of 1200 children, the study will have 80% power to detect at a 5% level of significance a 32% reduction in incidence of clinical attacks of malaria in children who receive chemoprevention during the surveillance period; 131 episodes of clinical malaria will be expected to occur in the control group followed until the end of one malaria transmission season. The sample size calculation was based on data from previous studies carried out in The Gambia and these studies used haematocrit to determine the presence of anaemia.

## **4.0 Ethics**

The study protocol and statement of informed consent will be approved by the Joint Gambia Government/MRC Ethics Committee and the London School of Hygiene and Tropical Medicine Ethics Committee. Only children whose parents/guardians give informed consent will be enrolled. The study involves collection of 5ml venous blood and fingerprick samples from sick children as part of routine care. In addition, one further fingerprick blood sample wwill be obtained from all children in the trial at the end of the malaria transmission season.

The study investigates an intervention appropriate to the health needs of The Gambia. The drug used for the trial is safe and is in routine use in The Gambia. Entry into the trial will not affect the health care the volunteers received.

## **4.1 Data Safety and Monitoring Board**

An independent committee consisting of experts in infectious diseases and biostatistics will be appointed to oversee ethical and safety aspects of the study. The DSMB will be informed of serious adverse experiences that occurred during the study (transmitted through the Local Safety Monitor). The DSMB will be empowered to put the study on hold pending review of potential safety issues. The protocol and the analytical plan will be reviewed and approved by the DSMB before they are implemented.

## **4.2 Local Safety Monitor**

A local safety monitor will be appointed. His role will be to support the clinical investigators and to act as a link between the investigators and the DSMB. All serious adverse events suspected to be related to trial medication will be reported to him in his capacity as a liaison to the DSMB.

## **5.0 Data management**

All baseline surveillance and laboratory data will be collected on forms designed for the trial. Field and laboratory staff will be trained to follow the procedures set out in a series of Standard Operating Procedures (SOPs).

.

**6.0 References**

1. Premji Z, Hamisi Y, Shiff C, Minjas J, Lubega P, Makwaya C. Anaemia and Plasmodium

falciparum infections among young children in an holoendemic area, Bagamoyo,

Tanzania. *Acta Trop* 1995;**59**(1)**:**55-64.

2. Newton CRJC, Warn PA, Winstanley PA, et al. Severe anaemia in children living in a

malaria endemic area of Kenya. *Tropical Medicine and International Health*

1997;**2**(2)**:**165-178.

3. Muhe L, Oljira B, Degefu H, Enquesellassie F, Weber MW. Clinical algorithm for malaria

during low and high transmission seasons. *Arch Dis Child* 1999;**81**(3)**:**216-20.

4. McElroy PD, ter Kuile FO, Lal AA, et al. Effect of Plasmodium falciparum parasitemia

density on hemoglobin concentrations among full-term, normal birth weight children in

western Kenya, IV. The Asembo Bay Cohort Project. *Am J Trop Med Hyg*

2000;**62**(4)**:**504-12.

5. May J, Falusi AG, Mockenhaupt FP, et al. Impact of subpatent multi-species and multiclonal

plasmodial infections on anaemia in children from Nigeria. *Trans R Soc Trop*

*Med Hyg* 2000;**94**(4)**:**399-403.

6. Abdulla S, Schellenberg JA, Nathan R, et al. Impact on malaria morbidity of a programme

supplying insecticide treated nets in children aged under 2 years in Tanzania:

community cross sectional study. *Bmj* 2001;**322**(7281)**:**270-3.

7. Lackritz EM, Campbell CC, Ruebush TK, 2nd, et al. Effect of blood transfusion on survival

among children in a Kenyan hospital. *Lancet* 1992;**340**(8818)**:**524-8.

8. Hedberg K, Shaffer N, Davachi F, et al. Plasmodium falciparum-associated anemia in

children at a large urban hospital in Zaire. *Am J Trop Med Hyg* 1993;**48**(3)**:**365-71.

9. Slutsker L, Taylor TE, Wirima JJ, Steketee RW. In-hospital morbidity and mortality due to

malaria-associated severe anaemia in two areas of Malawi with different patterns of

malaria infection. *Trans R Soc Trop Med Hyg* 1994;**88**(5)**:**548-51.

10. Marsh K, Forster D, Waruiru C, et al. Indicators of life-threatening malaria in African

children. *N Engl J Med* 1995;**332**(21)**:**1399-404.

36

11. Bojang KA, Van Hensbroek MB, Palmer A, Banya WA, Jaffar S, Greenwood BM.

Predictors of mortality in Gambian children with severe malaria anaemia. *Ann Trop*

*Paediatr* 1997;**17**(4)**:**355-9.

12. Schellenberg D, Menendez C, Kahigwa E, et al. African children with malaria in an area of

intense Plasmodium falciparum transmission: features on admission to the hospital

and risk factors for death. *Am J Trop Med Hyg* 1999;**61**(3)**:**431-8.

13. Alonso PL, Lindsay SW, Armstrong JR, et al. The effect of insecticide-treated bed nets on

mortality of Gambian children. *Lancet* 1991;337(8756):1499-502.

14. Greenwood BM, Greenwood AM, Snow, Byass P, Bennet S, Hatib-Njie AB, 1989. The effect of malaria chemoprophylaxis given by traditional birth attendants on the course and outcome of pregnancy. *Trans R Soc Trop Med Hy 83:589-594*

15. Brewster DR, Greenwood BM. Seasonal variation of paediatric diseases in The Gambia,

west Africa. *Ann Trop Paediatr* 1993;**13**(2)**:**133-46.

16. Snow RW, Bastos de Azevedo I, Lowe BS, et al. Severe childhood malaria in two areas of

markedly different falciparum transmission in east Africa. *Acta Trop* 1994;**57**(4)**:**289-

300.

17. Abdalla, S, Weatheral, D J., Wickramasinghe, S.N. & Hughes, M.. The anaemia of Plasmodium falciparum malaria. British Journal of Haematology 1980, 46, 171-183

18. Philips R.E. & Pasvol, G. Anaemia of *Plasmodium falciparum* malaria. Baillière’s Clinical Haematology 1992, 5, 315-330.

19. Weatheral, DJ, Abdalla S and Pippard MJ. The anaemia of *Plasmodium falciparum* malaria. Malaria and the Red Cell 1983. Ciba Foundation Symposium 94. Evered, D. & Whetan, J. (editor). London: Pitman, pp. 74-88

20. Menon A, Snow RW, Otoo LN, Greenwood BM. Decline in sensitivity of *Plasmodium falciparum* to chloroquine in The Gambia. Lancet 1987, 1:1029-30.

21. Menon A, Otoo LN, Herbage EA, Greenwood BM.. A national survey of the prevalence of chloroquine resistance to *Plasmodium falciparum* malaria in The Gambia. Transactions of the Royal Society of Tropical Medicine and Hygiene1990. 84: 638-640

22. Boele van Hensboroek M, Morris-Jones S Meissner S, Jaffar S, Bayo L, Dackour R, Philips C, Greenwood BM. Iron, but not folic acid, combined with effective antimalarial therapy promotes haemalological recovery in African children after an acute falciparum malaria. Transactions of the Royal Society of Tropical Medicine and Hygiene 1995, 89: 672-676

23. Müller O, Boele van Hensboroek M, Jaffar S, Drakeley C, Okorie C, Joof D, Pinder M, Greenwood BM. Tropical Medicine and International Health 1996, 1: 124-132.

24.von Seidlein L, Jawara M, Coleman R, Doherty T, Walraven G, Targett G. Parasitaemia and gametocytaemia after treatment with chloroquine, pyrimethamine/sulfadoxine, and pyrimethamine/sulfadoxine combined with artesunate in young Gambians with uncomplicated malaria. Trop Med Int Health. 2001, 6:92-8.
25*.* Lackritz EM. Prevention of HIV transmission by blood transfusion in the developing world:

achievements and continuing challenges. *Aids* 1998;**12**(Suppl A)**:**S81-6.

26. Bojang KA, Palmer A, Boele van Hensbroek M, Banya WA, Greenwood BM. Management

of severe malarial anaemia in Gambian children. Trans R Soc Trop Med Hyg

1997;91(5):557-61.

27. D’Alessandro U, Olaley B, McGuire W et al. Mortality and Morbidity from malaria in Gambian children after introduction of an impregnated bednet programme. Lancet 1995; 345: 479-83

28. Lengeler C. Insecticide treated bednets and curtains for malaria control (Cochrane Review). In: The Cochrane Library 1998; 3. Oxford: Update Sofware

29. Habluetzel A, Diallo D, Esposito F et al. Do insecticide impregnated curtains reduce all-cause mortality in Bukina Faso? Tropical Medicine and International Health 1997; 2(9): 855-62

30. Binka F, Kubaje A, Adjuik M et al. Impact of permethrin impregnated bednets on child mortality in Kassena-Nankana district Ghana: a randomised controlled trial. Tropical Medicine and International Health 1996; 1(2): 147-54

31. Abdulla S, Armstrong- Schellenberg J, Nathan R et al. Impact on malaria morbidity of a programme supplying insecticide treated nets in children aged under 2 years in Tanzania: community cross-sectional study. British Medical Journal 2001: 322: 270-73.

32. . Abdalla SH. Iron and folate status in Gambian children with malaria. *Ann Trop Paediatr*

1990;**10**(3)**:**265-72.

33. Bates CJ, Powers HJ, Lamb WH, Gelman W, Webb E. Effect of supplementary vitamins

and iron on malaria indices in rural Gambian children. *Trans R Soc Trop Med Hyg*

1987;**81**(2)**:**286-91.

34. Murray MJ, Murray AB, Murray MB, Murray CJ. The adverse effect of iron repletion on the

course of certain infections. *Br Med J* 1978;**2**(6145)**:**1113-5.

35. Oppenheimer SJ, Gibson FD, Macfarlane SB, Moody JB, Hendrickse RG. Iron

supplementation and malaria. *Lancet* 1984;**1**(8373)**:**389-90.

36. Oppenheimer SJ, Gibson FD, Macfarlane SB, et al. Iron supplementation increases

prevalence and effects of malaria: report on clinical studies in Papua New Guinea.

*Trans R Soc Trop Med Hyg* 1986;**80**(4)**:**603-12.

37. Oppenheimer SJ, Macfarlane SB, Moody JB, Bunari O, Hendrickse RG. Effect of iron

prophylaxis on morbidity due to infectious disease: report on clinical studies in Papua

New Guinea. *Trans R Soc Trop Med Hyg* 1986;**80**(4)**:**596-602.

38. Smith AW, Hendrickse RG, Harrison C, Hayes RJ, Greenwood BM. The effects on

malaria of treatment of iron-deficiency anaemia with oral iron in Gambian children.

*Ann Trop Paediatr* 1989;**9**(1)**:**17-23.

39. Harvey PW, Heywood PF, Nesheim MC, et al. The effect of iron therapy on malarial

infection in Papua New Guinean schoolchildren. *Am J Trop Med Hyg* 1989;**40:**12-8

40. Chippaux JP, Schneider D, Aplogan A, Dyck JL, Berger J. [Effects of iron

supplementation on malaria infection]. *Bull Soc Pathol Exot* 1991;**84**(1)**:**54-62.

41. Schneider D, Chippaux JP, Aplogan A, Dyck JL, Berger J. [Evaluation of the impact of

iron treatment. Interference of malaria]. *Bull Soc Pathol Exot* 1995;**88**(5)**:**260-4.

42. van den Hombergh J, Dalderop E, Smit Y. Does iron therapy benefit children with severe

malaria-associated anaemia? A clinical trial with 12 weeks supplementation of oral

iron in young children from the Turiani Division, Tanzania. *J Trop Pediatr*

1996;**42**(4)**:**220-7.

43. Oppenheimer SJ. Iron and its relation to immunity and infectious disease. *J Nutr*

2001;**131**(2S-2)**:**616S-633S; discussion 633S-635S.

44**.** Menendez C, Kahigwa E, Hirt R, et al. Randomised placebo-controlled trial of iron

supplementation and malaria chemoprophylaxis for prevention of severe anaemia

and malaria in Tanzanian infants . *Lancet* 1997;**350:**844-50.

45. Duraisingh MT, Curtis J, Warhurst DC. Plasmodium falciparum: detection of polymorphisms in the dihydrofolate reductase and dihydropteroate synthetase genes by PCR and restriction digestion. *Exp Parasitol* 1998;**89**(1)**:**1-8.

46. Djimde A, Doumbo OK, Cortese JF, Kayentao K, Doumbo S, Diourte Y, Dicko A, Su XZ, Nomura T, Fidock DA, Wellems TE, Plowe CV, Coulibaly D. A molecular marker for chloroquine-resistant falciparum malaria; N Eng J Med 2001; **344**: 257-301
47. WHO, Communicable Disease Cluster *(2000) Trans Roy Soc. Trop. Med. Hyg. 94 Supplement 1.*

48. Hans Verhoef, Clive E West, Silas M Nzyuko, Stefan de Vogel, Rikkert van der Valk, Mike A Wanga, Anneleen Kuijsten, Jacobien Veenemans, Frans J Kok. Intermittent administration of iron and sulfadoxine-pyrimethamine to control anaemia in Kenyan children: a randomised controlled trial. *Lancet* 2002, **360**: 908-14

**(d) Time-table**

Timetable

|  | 2003 | | | | | | | | | | | | 2004 | | | | | | | | | | | |
| --- | --- | --- | --- | --- | --- | --- | --- | --- | --- | --- | --- | --- | --- | --- | --- | --- | --- | --- | --- | --- | --- | --- | --- | --- |
|  | 1 | 2 | 3 | 4 | 5 | 6 | 7 | 8 | 9 | 10 | 11 | 12 | 1 | 2 | 3 | 4 | 5 | 6 | 7 | 8 | 9 | 10 | 11 | 12 |
| SCC & ERC submission |  | x | x |  |  |  |  |  |  |  |  |  |  |  |  |  |  |  |  |  |  |  |  |  |
| Enrolment |  |  |  |  |  |  | x | x | x | x | x | x |  |  |  |  |  |  | x | x | x | x | x | x |
| Morbidity surveillance |  |  |  |  |  |  | x | x | x | x | x | x |  |  |  |  |  |  | x | x | x | x | x | x |
| Mortality surveillance |  |  |  |  |  |  | x | x | x | x | x | x |  |  |  |  |  |  | x | x | x | x | x | x |
| Monthly SP treatment |  |  |  |  |  |  | x | x | x | x | x | x |  |  |  |  |  |  | x | x | x | x | x | x |
| End of season survey |  |  |  |  |  |  |  |  |  |  |  | x |  |  |  |  |  |  |  |  |  |  |  | x |
| Dry season visit |  |  |  |  |  |  |  |  |  |  |  |  |  |  |  |  | x |  |  |  |  |  |  |  |

Dose 1 of SP/ placebo

Hb &BF

Monthly SP/placebo at hospital/HC

Passive surveillance, Hb & BF

Clinical assessment

BF & Hb

Clinical assessment, treatment,

± transfusion, Hb & BF

Enrolment, admission

Discharge from Hospital/ HC

Monthly hospital/HC visit

Hospital/HC visit if unwell

Day 7 OPD

visit

End of malaria season

survey

**Figure 4-2: Flow chart of study activities**
